# Genome Sequence of a Novel Polymer-Grade l-Lactate-Producing Alkaliphile, *Exiguobacterium* sp. Strain 8-11-1

**DOI:** 10.1128/genomeA.00616-13

**Published:** 2013-08-15

**Authors:** Xu Jiang, Yanfen Xue, Limin Wang, Bo Yu, Yanhe Ma

**Affiliations:** CAS Key Laboratory of Microbial Physiological and Metabolic Engineering, Institute of Microbiology, Chinese Academy of Sciences, Beijing, Chinaa; State Key Laboratory of Microbial Resources, Institute of Microbiology, Chinese Academy of Sciences, Beijing, Chinab; Tianjin Institute of Industrial Biotechnology, Chinese Academy of Sciences, Tianjin, Chinac

## Abstract

*Exiguobacterium* sp. strain 8-11-1 is a newly isolated alkaliphile, which was reported to efficiently produce l-lactate using NaOH as the neutralizing agent. Here, we present the first 2.9-Mb assembly of its genome sequence, which may provide useful information related to its efficient lactate production and sodium ion tolerance capacities.

## GENOME ANNOUNCEMENT

The genus *Exiguobacterium* was first described in 1983 by Collins et al. (1), with the characterization of the type species *Exiguobacterium aurantiacum*. In addition to the type strains, other *Exiguobacterium* spp. have been isolated from or molecularly detected in a wide range of habitats, including cold and hot environments with temperatures ranging from -12°C to 55°C (2). Many organisms of the genus *Exiguobacterium* are also haloalkaliphiles (3, 4). Therefore, they have properties of biotechnological interest (5, 6). *Exiguobacterium* sp. strain 8-11-1, an alkaliphile isolated from soil collected in a salt lake in Inner Mongolia, China, was demonstrated to be a good producer of l-lactic acid from glucose by using NaOH as the neutralizing agent. The high levels of optically pure l-lactic acid produced by *Exiguobacterium* sp. 8-11-1, combined with the ease of handling and low costs associated with the open fermentation strategy, provide a novel and potentially important approach for future l-lactic acid production (7).

Here, we present the first draft genome sequence of *Exiguobacterium* sp. 8-11-1, which was obtained by using the Illumina HiSeq 2000 system (300-bp paired-end sequences), which was performed by the Chinese National Human Genome Center at Shanghai, China. A total of 5,212,033 high-quality read pairs were produced for *de novo* assembly using the Velvet program (8). The reads were assembled into 61 contigs, providing 369-fold coverage. The contig N_50_ is 127,764 bp and the largest contig assembled was 345,491 bp. The average length of assembled contigs is 47,655 bp, with a total length of 2,906,962 bp.

Gene prediction and genome annotation were performed by the RAST server and the NCBI PAPPC (9, 10). tRNAs were predicted using the tRNAscan software (11). The gene function and classification were performed using the KEGG and Clusters of Orthologous Groups (COG) databases (12). About 2,926 coding sequences (CDSs) (average length, 887 bp), with a G+C content of 54%, were predicted, including 2,165 proteins having identified functions. Fourteen tRNA and 4 rRNA genes were also annotated. The metabolic network of 8-11-1 (determined by RAST) was reconstructed (9). *Exiguobacterium* sp. 8-11-1 is predicted to possess complete metabolic pathways, including those for glycolysis, the tricarboxylic acid cycle, and the pentose phosphate pathway. In addition, *Exiguobacterium* sp. 8-11-1 has the genes encoding xylose isomerase, l-lactate dehydrogenase, and permease, which indicates that strain 8-11-1 is a potential producer of l-lactic acid from cellulosic substrate. There are 134 genes for inorganic ion transport and metabolism, including 1 MnhB-type Na^+^/H^+^ antiporter and 5 NhaC-type Na^+^/H^+^ antiporters, which may contribute to pH homeostasis and the capacity of the cells to lower the cytoplasmic Na^+^ concentration optimally at the alkaline pH (13). The detailed analysis of the genomic information of strain 8-11-1 will provide further insights into the genetic versatility of *Exiguobacterium* strains, as well as their extremophilic properties.

### Nucleotide sequence accession numbers.

This whole-genome shotgun project has been deposited at DDBJ/EMBL/GenBank under the accession no. ATKK00000000. The version described in this paper is version ATKK01000000.
